# Leaf Anatomy and Photochemical Behaviour of *Solanum lycopersicum* L. Plants from Seeds Irradiated with Low-LET Ionising Radiation

**DOI:** 10.1155/2014/428141

**Published:** 2014-04-23

**Authors:** V. De Micco, R. Paradiso, G. Aronne, S. De Pascale, M. Quarto, C. Arena

**Affiliations:** ^1^Department of Agricultural and Food Sciences, University of Naples Federico II, via Università 100, 80055 Portici, Italy; ^2^Department of Physics, University of Naples Federico II, Via Cinthia, 4-80126 Naples, Italy; ^3^Department of Biology, University of Naples Federico II, Via Cinthia, 4-80126 Naples, Italy

## Abstract

Plants can be exposed to ionising radiation not only in Space but also on Earth, due to specific technological applications or after nuclear disasters. The response of plants to ionising radiation depends on radiation quality/quantity and/or plant characteristics. In this paper, we analyse some growth traits, leaf anatomy, and ecophysiological features of plants of *Solanum lycopersicum* L. “Microtom” grown from seeds irradiated with increasing doses of X-rays (0.3, 10, 20, 50, and 100 Gy). Both juvenile and compound leaves from plants developed from irradiated and control seeds were analysed through light and epifluorescence microscopy. Digital image analysis allowed quantifying anatomical parameters to detect the occurrence of signs of structural damage. Fluorescence parameters and total photosynthetic pigment content were analysed to evaluate the functioning of the photosynthetic machinery. Radiation did not affect percentage and rate of seed germination. Plants from irradiated seeds accomplished the crop cycle and showed a more compact *habitus*. Dose-depended tendencies of variations occurred in phenolic content, while other leaf anatomical parameters did not show distinct trends after irradiation. The sporadic perturbations of leaf structure, observed during the vegetative phase, after high levels of radiation were not so severe as to induce any significant alterations in photosynthetic efficiency.

## 1. Introduction

The effect of ionising radiation on plants is studied within different frameworks. On one hand, high levels of radiation are an undesirable factor affecting plant growth in Space or on Earth in case of nuclear disasters, such as those of Chernobyl and Fukushima [1, 2]. On the other hand, ionising radiation is applied as a tool to induce a wide spectrum of mutations in breeding programs or as means for microbial decontamination [3, 4]. Although focusing on different types of radiation and on different biological endpoints, most experiments aiming to highlight the response of plants to ionising radiation have a common aspect: they analyse the effect of a single type of radiation with wide range of doses in order to build dose-response curves and to test plant radioresistance [2]. Generally, the response to low doses (i.e., up to 10 Gy) is interesting for Space-related issues; the use of increasingly higher doses is important not only for their role as positive controls (plant reaction being more likely expected at high doses), but also to generate information useful for terrestrial applications of radioecology [2, 5]. Focusing on Space exploration, there is common agreement that long-term human permanence in Space relies on the possibility to regenerate resources in Bioregenerative Life Support Systems (BLSSs) where plants can play a key role [6, 7]. Plant growth is not prevented in Space; however, although plants can be more resistant than other organisms to specific Space factors (including reduced gravity and cosmic radiation), there is also evidence of altered growth and reduced fitness [2, 8, 9]. Many studies have indicated the occurrence of either positive or negative phenomena in plants exposed to low-LET (linear energy transfer) (e.g., X- and gamma-rays) and high-LET (e.g., protons and heavy ions) ionising radiation [2]. Definitely, in many experiments carried out in Space, plants were exposed to various combinations of both altered gravity and radiation. Ground-based research helps distinguish between plant reactions triggered by each one of the two factors. On Earth, the simulation of the complex Space radiation spectrum, including the radiation emitted from the Sun and galactic cosmic rays, is impossible, even though single particles can be produced at large particle accelerators [10]. The exposure likely to be encountered by organisms in Space depends on the shielding features of the space vehicles and/or planetary platforms as well as on the evolution of the solar cycle during the permanence in Space; however, it can be estimated that during a relatively quiet solar cycle, a permanence of one year in Space would expose organisms to a dose of less than 10 Gy [10–12]. In Space, a large fraction of the dose is delivered by protons (approximately 87%), which are low-LET particles with similar effectiveness compared to X-rays. Therefore, initial studies can exploit conventional X-rays as the reference radiation to obtain data which are the necessary basis for future comparison using accelerated ion beams [13, 14]. The severity of the effects of low- and high-LET ionising radiation depends on several factors related to the radiation itself (e.g., type, total dose, and dose rate) and to plant features (e.g., species, cultivar, plant age, complexity of the target organ or tissue, and level of ploidy) [2, 15]. An interesting phenomenon in plants, known as* hormesis*, is the occurrence of positive responses to low doses of ionising radiation which would stimulate processes such as germination and growth [16]. A recent paper by Marcu et al. [17] showed enhanced germination, plant growth, and synthesis of photosynthetic pigments when seeds were irradiated with low doses of gamma rays, but opposite responses when doses were high. Sparsely and densely ionising radiation can also determine mutations inducing favourable agronomical traits such as earlier maturity, higher yields, and resistance to diseases [18, 19]. On the other hand, there is evidence of altered gene expression due to plants' exposure to ionising radiation leading to detrimental consequences culminating in plant death [20].

The very prolific literature on radiation-induced genetic aberrations and metabolic alterations (especially linked to the ROS—reactive oxygen species—production) is accompanied by scarce information at the structural level [2]. Given that the physics of plant structure ultimately regulate major metabolic and physiological processes [21], the development of well-structured above-ground organs is needed to obtain efficient photosynthesis. The photosynthetic process can be considered one of the main functions accomplished by higher plants in plant-based modules of BLSSs for air regeneration. Thus, knowing how ionising radiation affects morphogenesis, and even more leaf development, is interesting to evaluate possible impairment in photosynthesis which would constrain the maximisation of resource efficiency in the BLSSs. Indeed, the optimisation of resource use in the BLSSs is considered a challenge in Space exploration [6, 22–24].

Space factors can directly alter the inner structure of plants or impair photosynthesis due to the alteration of gas exchange or of functioning of the photosystems. Indeed, the lack of convective forces due to microgravity can alter the availability of gases at the carboxylation sites [25]. On the other hand, high levels of ionising radiation can negatively affect pigment-protein complexes and enzymes responsible for light absorption, electron transport, and carbon reduction cycle [2]. Moreover, high radiation levels may also induce photoprotection mechanisms and affect the behaviour of the D1 protein in the PSII repair cycle [26, 27]. Photoprotection mechanisms can rely on physiological adjustments [14, 28] and/or on the intensification of structural and biochemical barriers such as trichomes and phenolic compounds acting as natural screens against radiation [29]. Indeed, the increased content of phenolic compounds linked to chloroplast membranes has been shown in bean leaves irradiated with high doses of X-rays [30].

The main aim of this paper is to analyse whether X-ray irradiation of* Solanum lycopersicum* L. “Microtom” at the seed stage affects leaf development and anatomy, fluorescence parameters, and pigment content. This study has been conducted within a wider experimentation aiming to analyse the effect of irradiation performed at seed stage on various growth processes of dwarf tomato. In this paper, we focus on the analysis of leaf morphofunctional traits in two leaf types. In particular, we analyse leaves maintaining juvenile traits (i.e., the first two true leaves characterised by simpler morphology than successive leaves) and adult compound leaves, in order to evaluate whether similar structures characterised by different complexity and age show differential responses. Indeed, it is recognised that the increase in morphological complexity confers more efficient buffering capacity to biological systems for dealing with radiation-induced damage [31]. An X-ray source at 250 kV was used to irradiate the seeds because X-rays constitute the reference radiation to assess the damage caused by any other radiation source at the same dose. The dwarf cultivar Microtom was selected as plant material because it is a model system widely used for research in molecular biology [32–34]. Moreover, its compact habit, short life cycle, and fruit development, not requiring hand pollination, are desirable features in BLSSs and would make this cultivar a model system also for research in plant Space biology.

## 2. Materials and Methods

### 2.1. Plant Material and Irradiation Procedure

The experiment was conducted using seeds of* Solanum lycopersicum* L. “Microtom” provided by Holland Online Vof (Amsterdam, The Netherlands). Seed irradiation was performed on April 2013. Dry seeds were placed into Petri dishes in one layer and irradiated with five doses of X-rays (0.3 Gy, 10 Gy, 20 Gy, 50 Gy, and 100 Gy) 250 kVp, at dose rate of 1 Gy/min. A set of three Petri dishes with 15 seeds each was used for each irradiation treatment and for a nonirradiated control. Doses up to 20 Gy were chosen in order to build a reference-response range to explore plant sensitivity, as generally set in experiments to evaluate the effect of radiation on biological systems [13, 14, 28, 30]. The highest doses (50 Gy and 100 Gy) are generally applied since they can be considered as positive controls, as there is more likely a plant reaction at these doses. X-rays were delivered as one dose per each Petri dish [35], in order to avoid cumulative effects on the same sample. X-rays were produced by a Thomson tube (TR 300 F, 250 kVp, Stabilipan, Siemens, Forchheim, Germany) with tungsten cathode, filtered by 1 mm thick copper foil and with 15 mA anodic current. Before irradiation, a physical dosimetry was performed by using an ionization chamber (Victoreen, Mödling, Austria). The X-ray intensity was measured at the established distance where seeds were positioned under the X-ray tube.

After the exposure to X-rays (on 24 April), the irradiated seeds and controls were placed into Petri dishes on three layers of filter paper moistened with distilled water. Petri dishes were incubated in the dark at temperature of 20°C and monitored daily to analyse germination percentage and rate (considered as the number of days taken to reach the maximum germination percentage). Seeds were classified as germinated when the emerging root grew as long as the seed maximum diameter.

### 2.2. Growth Conditions

The growth experiment was carried out in a greenhouse with a polyethylene film cover. The greenhouse was equipped with a black plastic shading net (70% shading), in order to keep the temperature and light intensity close to the levels feasible in test bed evaluation of BLSSs technologies [22, 23]. Wavelength composition of the sunlight, including the whole PAR range spectrum for photosynthetic activity, was not affected.

Seedlings were transplanted into pots (4 cm diameter) on peat-based compost (peat : soil, 1 : 1 in volume) and transferred to the greenhouse, 5 days after sowing (DAS). Then, they were repotted in 10 cm pots using the same substrate, at the stadium of fourth true leaf (i.e., two juvenile and two compound leaves) at 22 DAS. In the cultivar Microtom, the first two true leaves maintain juvenile traits and are characterised by simple morphology: they are trifoliate with little or not evident lobes. Successive leaves show more complex morphology, having pennate-compound lamina with highly indented lobes.

During the whole growth period, plants were irrigated with tap water at 2-day interval in order to reach the container capacity (till the beginning of drainage).

The temperature inside the greenhouse was recorded every 10 minutes by means of a data logger (Tynitag Ultra2, Gemini Data Loggers, Chichester, UK). Photosynthetic photon flux density (PPFD) at the canopy level was recorded daily at 12.00 p.m., with a Delta OHM DO9847 multifunction meter.

The whole crop cycle (up to fruit ripening) lasted from 24 April until 6 August 2013.

During the whole growth cycle, the mean values of temperature inside the greenhouse were 28.4/21.6°C (day/night) and the light intensity recorded at noon at the canopy level was 446 *μ*mol m^−2^ s^−1^ on average.

### 2.3. Biometric and Microscopy Analyses

Biometric analyses were performed once a week on 10 plants per treatment throughout the crop cycle. Plant height and number of leaves were recorded. Moreover, single leaves of the main stem were photographed with a digital camera (Nikon D3100, Nikon Europe B.V.) in order to examine leaf area increments with the software program AnalySIS 3.2 (Olympus, Hamburg, Germany).

From 5 plants per treatment, the second juvenile leaf (JL) and the first compound leaf (CL) were collected when they were fully developed. One median leaflet (one of the two opposite leaflets below the apex) per leaf was used for microscopy analyses, while the others were used for pigment extraction.

Leaflets destined to microscopy analyses were immediately fixed in FAA (40% formaldehyde/glacial acetic acid/50% ethanol-5/5/90 by volume) for several days. Leaflets were cut under a dissection microscope (ZSX9, Olympus) to obtain subsamples of 5 × 5 mm in the middle of lamina, including the main vein. Subsamples were dehydrated in an ethanol series and embedded in the acrylic resin JB4 (Polysciences, Warrington, PA, USA). Semithin cross sections (5 *μ*m thick) were cut through a rotative microtome. Sections were stained with 0.025% Toluidine blue in 0.1 M citrate buffer at pH 4 [36], mounted with Canadian Balsam, and observed under a light microscope (BX60, Olympus). Unstained sections were mounted with mineral oil for fluorescence and observed under an epifluorescence microscope (BX60, Olympus) equipped with a mercury lamp, band-pass filter 330–385 nm, dichromatic mirror 400 nm and above, and barrier filter 420 nm and above. With these filters it was possible to detect the presence of simple phenolics that are autofluorescent at such wavelengths [37, 38]. Three sections per leaflet were analysed and images were collected by means of a digital camera (CAMEDIA C4040, Olympus) at various magnifications.

### 2.4. Digital Image Analysis

All digital images were analysed with the AnalySIS 3.2 (Olympus) software for image analysis. The thickness of the palisade and the spongy parenchymas were measured in five regions along the leaf lamina. The cell area and shape of upper and lower epidermis, palisade, and spongy parenchyma were quantified in 15 cells per each tissue per section. More specifically, cell shape was characterised as (a) aspect ratio (maximum width/height ratio of a bounding rectangle for the cell, defining how it is elongated), (b) sphericity (roundness of a particle with spherical particles having a maximum value of 1), and (c) convexity (the fraction of the cell's surface area and the area of its convex; a turgid cell has a maximum value of 1) [39, 40]. The cell area occupied by phenolic compounds was measured in 3 regions (150 × 200 *μ*m^2^ each) of the mesophyll per section selected avoiding veins. Thus, the area occupied by phenolic compounds was calculated as the percentage of tissue/picture occupied by compounds appearing autofluorescent at the above-reported filter settings [41].

### 2.5. Fluorescence Measurements and Pigment Content

Fluorescence measurements and pigment extraction were conducted on 5 pennate-compound leaves of 5 plants subjected to each irradiation treatment and in controls at 40 DAS.

Chlorophyll a fluorescence measurements were carried out by means of a pulse amplitude modulated fluorometer (Junior-PAM, Walz, Germany), equipped with a monitoring Leaf-Clip JUNIOR-B (Walz, Germany). On 30 min dark-adapted leaves, the background fluorescence signal, Fo, was induced by internal light provided by a blue LED of about 2-3 *μ*mol photons*·*m^−2^·s^−1^, at a frequency of 0.5 kHz. The maximal fluorescence level in the dark-adapted state (Fm) was measured by 1 s saturating light pulse (10 000 *μ*mol photons·m^−2^·s^−1^) at a frequency of 10 kHz; the maximal PSII photochemical efficiency (Fv/Fm) was calculated as Fv/Fm = (Fm − Fo)/Fm. The measurements in the light were carried out by exposing each leaf to a PPFD of 420 *μ*mol photons·m^−2^·s^−1^ for 5 min. This level of PPFD was chosen because it falls in the range of maximal quantum yield for the Microtom cultivar as indicated by fluorescence fast-response curves to light (data not shown). The steady-state fluorescence signal (Ft) and the maximal fluorescence (Fm′) under illumination were measured, setting the light measure at a frequency of 10 kHz. Fm′ was determined by a 1 s saturating light pulse (10 000 *μ*mol photons·m^−2^·s^−1^).

The quantum yield of the PSII electron transport (ΦPSII) and nonphotochemical quenching (NPQ) were expressed according to Genty et al. [42] and Bilger and Björkman [43].

After fluorescence determinations, leaves were collected from plants for the photosynthetic pigment content determination, namely, chlorophylls and carotenoids. Pigments were extracted with a mortar and pestle in ice-cold 100% acetone and quantified by a spectrophotometer (UV-VIS Cary 100, Agilent Technologies, USA) according to Lichtenthaler [44].

### 2.6. Statistical Analysis

All results were subjected to statistical analysis using the SPSS statistical package (SPSS Inc., Chicago, IL, USA). For the time course of plant height, number of leaves, and leaf area, interpolation equations (second-order polynomial curve) were calculated. For anatomy and ecophysiology, data were subjected to ANOVA; multiple comparison tests were performed with Student-Newman-Keuls and Duncan coefficients using *P* < 0.05 as the level of probability. Data on sphericity, convexity, and percentage of phenolics were transformed through arcsine function before statistical analysis.

## 3. Results

### 3.1. Effect of Ionizing Radiation on Seed Germination

Exposure of dry seeds to X-rays did not affect their percentage of germination which ranged between 97.8 and 100%, with no significant differences among treatments. Similarly, X-rays did not determine significant alterations in germination rates which ranged between 5.0 and 6.7 days.

### 3.2. Effect of Ionizing Radiation on Plant Growth

**Figure 1:**
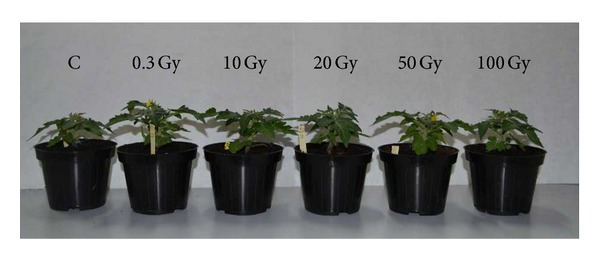
Plants of* S. lycopersicum* L. “Microtom” developed from control and irradiated seeds at 40 DAS (Days After Sowing). Plants from seeds exposed to increasing doses of X-rays (from 0 to 100 Gy) are shown from left to right.

Plants from both control and irradiated seeds completed their crop cycle up to fruit ripening in about 100 DAS; plants from irradiated seeds did not show any apparent alteration in above-ground growth (Figure 1).

**Figure 2:**
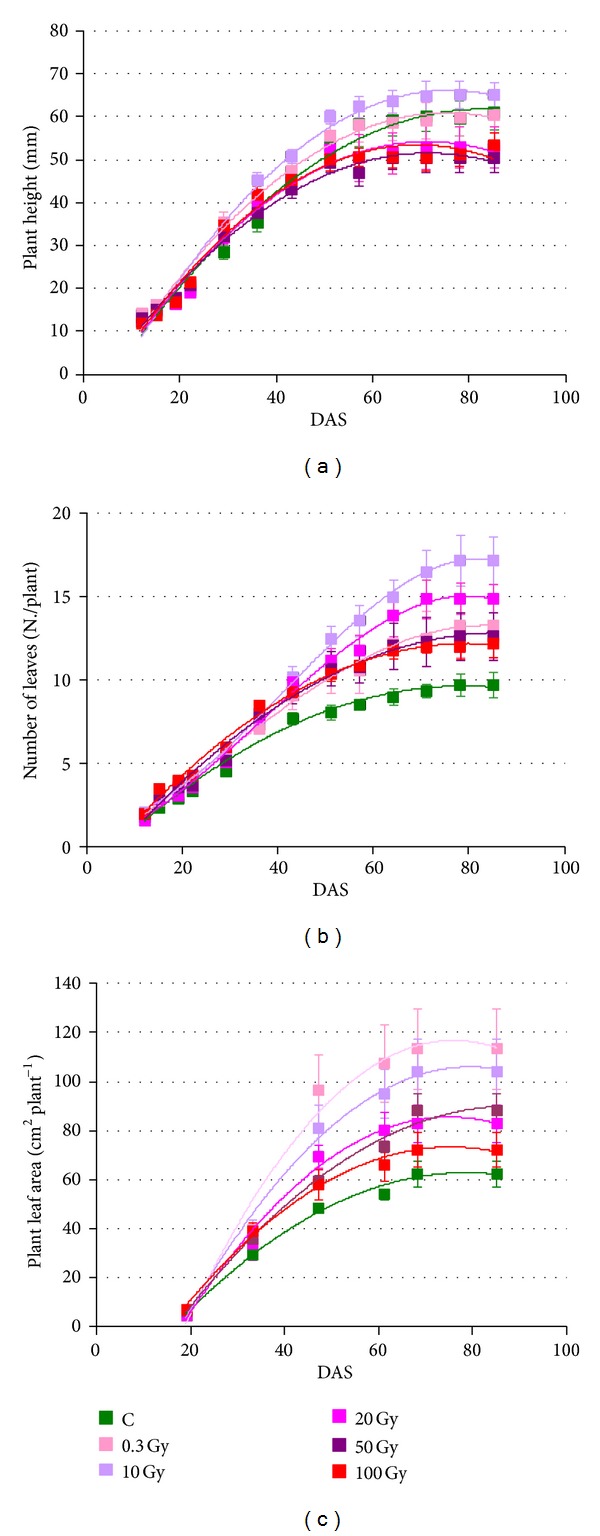
Time course of the plant height (a), number of leaves (b), and plant leaf area (c) in* S. lycopersicum* L. “Microtom” as a function of the X-ray doses applied at the seed stage. Mean values and standard errors are shown (*n* = 5). All second order polynomial curves had *r* ^2^ values above 0.97.

Figure 2 shows the effects of the seed irradiation treatments on plant height, leaf number, and plant leaf area of the main stem. The irradiation dose differently affected these growth parameters. In plants of the nonirradiated control, plant height reached the maximum value of 60.2 cm at approximately 71 DAS. Irradiation of seeds with 0.3 Gy did not significantly influence plant height. Starting from 71 DAS until the end of the cycle, height of plants from seeds irradiated with 10 Gy was significantly higher than that reached after irradiation with 20, 50, and 100 Gy. Final height of control plants and of those from seeds irradiated with X-ray doses up to 10 Gy was significantly higher than that reached after irradiation with 20 Gy and above (Figure 2(a)).

The progression of the number of leaves and leaf area were not consistent with the time course of plant height (Figure 2). Indeed, in the control, at the end of the crop cycle, plants were characterized by 9.7 leaves, corresponding to 62.5 cm^2^ leaf area. Plants from irradiated seeds formed a number of leaves significantly higher than control at the end of the cycle, with maximum value in plants from seeds irradiated with 10 Gy (Figure 2(b)). Similarly, under all the radiation doses but 100 Gy, plants showed significantly larger leaf area than the control with maximum value at 0.3 Gy (Figure 2(c)).

### 3.3. Effect of Ionizing Radiation on Leaf Anatomy

**Figure 3:**
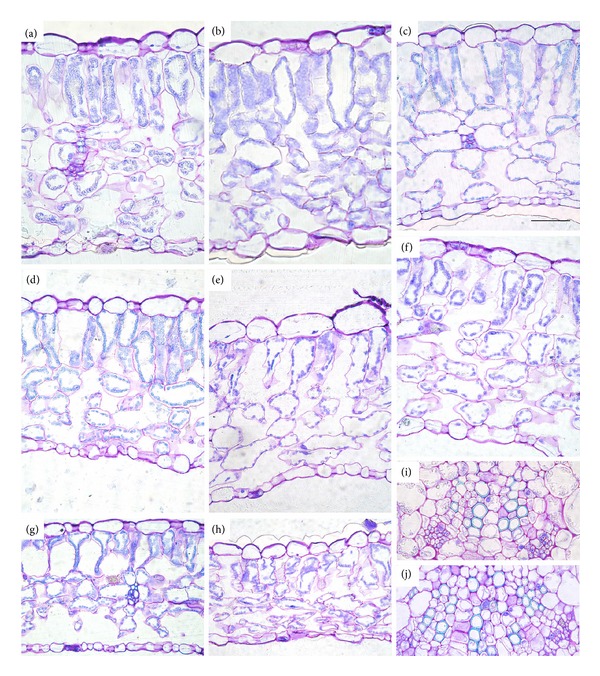
Light microscopy views of cross sections of leaf lamina of* S. lycopersicum *L. “Microtom” compound leaves from plants developed from control (a) and irradiated seeds: 0.3 Gy (b), 10 Gy (c), 20 Gy (d), 50 Gy (e, g), and 100 Gy (f, h). Detail of xylem in the main vein is shown in leaves from control (i) and 100 Gy irradiated seeds (j). Images (a–h) are at the same magnification. Bar = 50 *μ*m.

**Figure 4:**
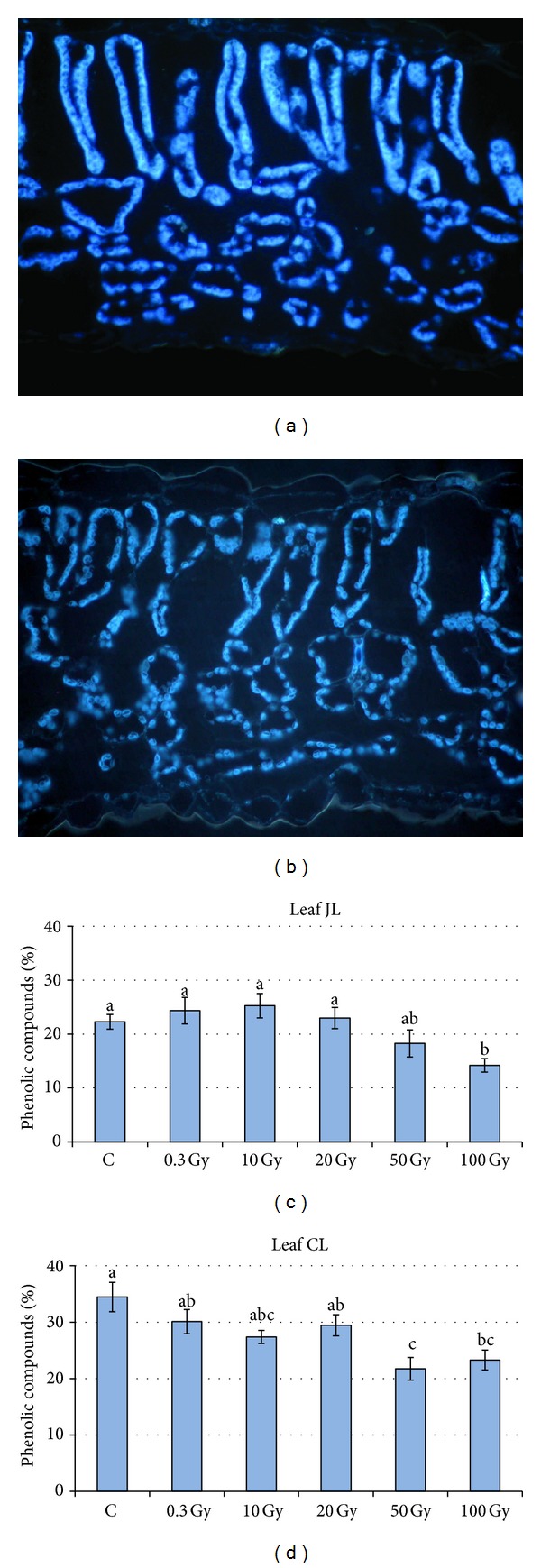
Epifluorescence microscopy views of lamina cross sections of* S. lycopersicum* L. “Microtom” compound leaves from plants developed from control (a) and 100 Gy irradiated seeds (b). Phenolic compounds along chloroplast membranes are autofluorescent. Percent of mesophyll occupied by phenolic compounds (%) is shown in (c) and (d) for juvenile (JL) and compound (CL) leaves, respectively. Mean values and standard errors are reported (*n* = 15); different letters correspond to significantly different values after multiple comparison tests (*P* < 0.05).

Microscopy analysis of the leaf sections showed a regular anatomical organisation in both juvenile and compound leaves formed after seed irradiation at different levels of X-rays. Since the effect of the treatments was similar in both types of leaves, microscopy views are shown only for compound leaves (Figure 3). Both leaf types were characterised by a typical dorsiventral structure which did not face disruptive structural alterations after irradiation of seeds with X-rays. Microscopy observations of leaves from seeds irradiated with up to 20 Gy did not reveal evident differences compared with control leaves (Figures 3(a)–3(d)). At 50 and 100 Gy, sporadic perturbations in the mesophyll structure were detectable (Figures 3(e)–3(h)). More specifically, the presence of more shrunk mesophyll cells (Figures 3(g) and 3(h)), due to palisade cells collapsed in the lower portion (Figure 3(g)) and spongy cells characterised by more angular shape (Figure 3(h)), was found. The occurrence of irregular-shaped cells of leaves from seeds irradiated with 50 and 100 Gy was confirmed by significant lower convexity values than control and low-dose treatments (Tables 1 and 2). The epidermis of compound leaves from irradiated seeds tended to have less elongated cells (i.e., lower aspect ratio and higher sphericity), while in the palisade parenchyma an opposite trend was observed (Table 2). No specific tendency of cell shape variation was found in the juvenile leaves (Table 1). The main veins did not show any irregularities in vessel walls after any irradiation treatments (Figures 3(i) and 3(j)). Epifluorescence microscopy showed the autofluorescence of phenolic compounds which were mainly localised along chloroplast membranes in both control leaves and those from irradiated plants (Figures 4(a) and 4(b)). No differences in autofluorescence distribution were observed in mesophyll cells of juvenile and compound leaves from control and irradiated seeds. The percentage of phenolic compounds showed a nonsignificant tendency to increase in juvenile leaves at low irradiation doses, followed by a significant decrease at 50 and 100 Gy (Figure 4(c)). In compound leaves, phenolic compounds tended to decrease after all irradiation treatments, with the lowest values in leaves from seeds irradiated at 50 and 100 Gy (Figure 4(d)).

**Figure 5:**
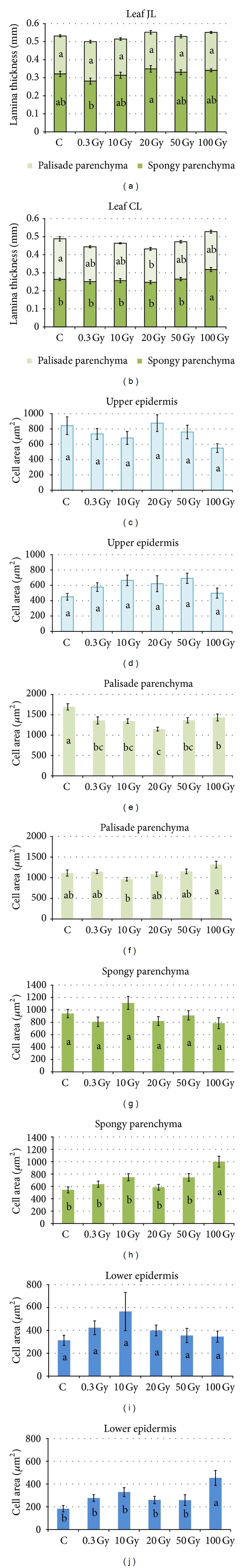
Lamina thickness (a, b) and cell area of upper epidermis (c, d), palisade (e, f), spongy (g, h) parenchymas, and lower epidermis (i, j) of* S. lycopersicum *L. “Microtom” leaves of control and irradiated plants at different doses of X-rays in both juvenile (JL) and compound (CL) leaves. Mean values and standard errors are shown (*n* = 15 in (a, b); *n* = 45 in (c–j)). Different letters correspond to significantly different values according to multiple comparison tests (*P* < 0.05).

The other analysed anatomical parameters did not show a clear-cut dose-dependent response (Figure 5; Tables 1 and 2). In juvenile leaves, the size of cells significantly decreased after the irradiation of seeds only in palisade parenchyma, with the lowest values at 20 Gy (Figures 5(c), 5(e), 5(g), and 5(i)). By contrast, in compound leaves, the application of 100 Gy resulted in increased cell size compared with control and leaves from seeds irradiated up to 50 Gy (Figures 5(d), 5(f), 5(h), and 5(j)).

### 3.4. Effect of Ionizing Radiation on Fluorescence Measurements and Pigment Content

**Figure 6:**
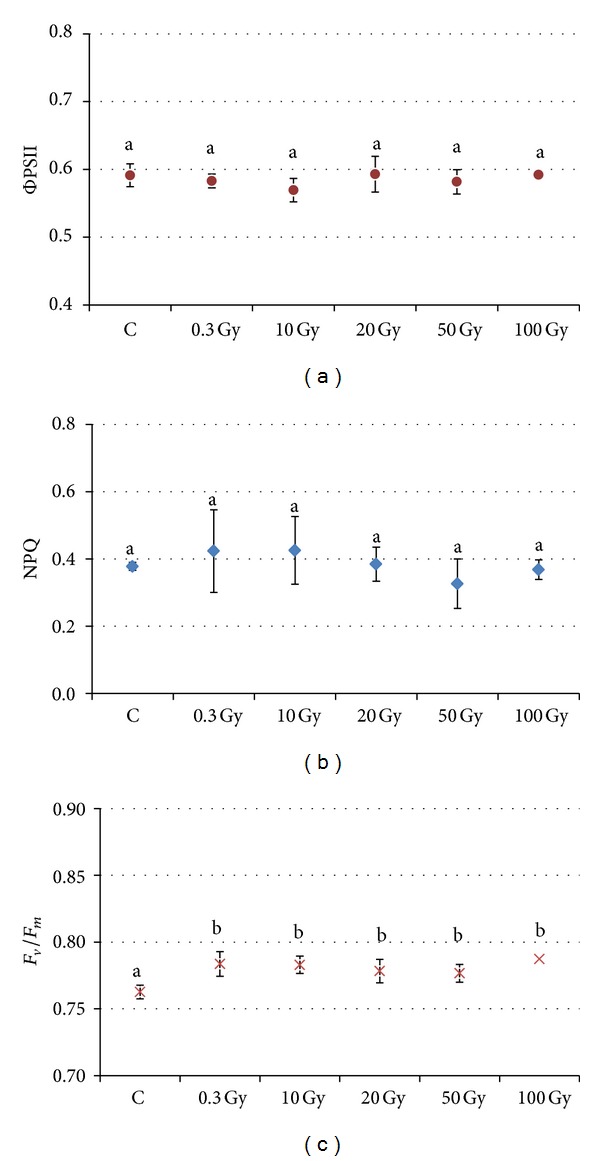
Quantum yield of PSII electron transport (ΦPSII, a), nonphotochemical quenching (NPQ, b), and maximal PSII photochemical efficiency (Fv/Fm, c), in compound (CL)* S. lycopersicum *L. “Microtom” leaves of control and plants from seeds irradiated at different doses of X-rays. Mean values and standard deviations are shown (*n* = 5). Different letters correspond to significantly different values according to multiple comparison tests (*P* < 0.05).

No significant differences were detectable in fluorescence parameters and total photosynthetic pigment content in plants grown from irradiated seeds at different X-rays doses (Figure 6). A tendency to NPQ increase was found at 0.3 and 10 Gy, followed by a decrease at doses higher than 20 Gy. However, the lack of significant differences in ΦPSII and NPQ among treatments indicated that X-rays, delivered at the seed stage on tomato plants, did not alter the capability of photosynthetic apparatus of tomato plants either to convert light energy in photochemistry or to perform thermal dissipation mechanisms. The maximum photochemical efficiency of PSII (Fv/Fm) was higher in leaves from irradiated seeds than control and did not show variations among different X-rays treatments; this indicated the maintenance of higher level of photosynthetic activity after irradiation, irrespectively from dose (Figure 6(c)).

**Figure 7:**
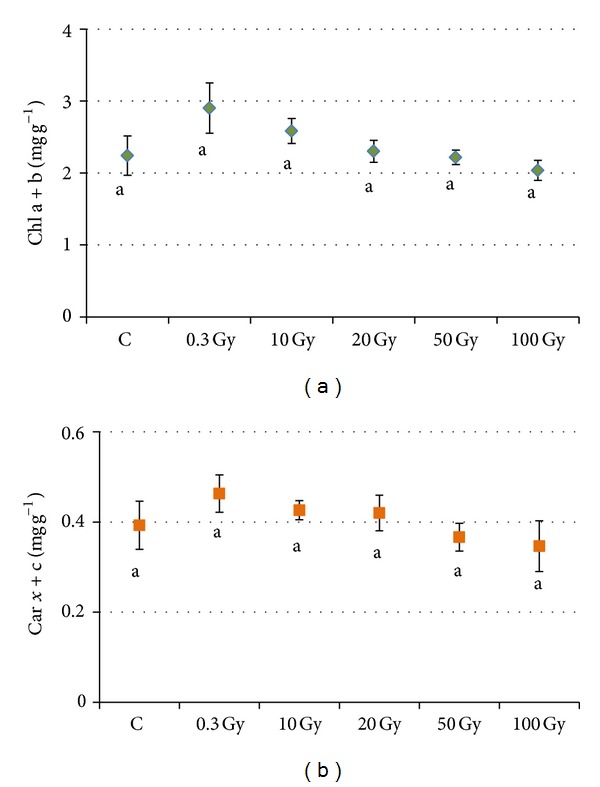
Total chlorophyll content (Chl a+b) (a) and total carotenoid content (Car x+c) (b), in compound (CL)* S. lycopersicum *L. “Microtom” leaves of control and plants from seeds irradiated at different doses of X-rays. Mean values and standard errors are shown (*n* = 5). Different letters correspond to significantly different values according to multiple comparison tests (*P* < 0.05).

The total chlorophyll, as well as the total carotenoid content, did not show significant changes due to irradiation of seeds (Figure 7).

## 4. Discussion

Ionising radiation can have positive, null, or negative effects on plant development depending on the properties of the radiation, the features of the plant material, and possible interactions with other environmental factors [2]. There is common agreement that plants are characterised by high radioresistance; moreover, low-LET ionising radiation has been reported to increase germination percentage, root length, plant height, and other growth parameters especially at low doses which induce* hormesis* [16, 17, 45–47]. Our results suggest that* S. lycopersicum* “Microtom” is characterised by high radioresistance at the seed stage: indeed, irradiation of dry seeds with a wide range of X-ray doses did not hamper germination and plant growth up to the completion of the crop cycle. An* hormesis* effect was evident since growth either remained unaffected or was positively affected especially by low doses of X-rays. Apart from plants grown from seeds irradiated with 0.3 and 10 Gy, all plants from irradiated seeds were characterised by a more compact* habitus*: higher number of larger leaves was not accompanied by increased height. The radiation-induced more compact growth pattern is positive in BLSSs in Space where availability of volume for growth is a major technical constraint [7, 48, 49]. In the cultivar Microtom, irradiation with doses equal to or higher than 20 Gy caused a decrease in plant height, in agreement with the induction of dwarf or semidwarf growth reported as a general effect of exposure to ionising radiation [18]. Hence, irradiation on the “Microtom” seeds would intensify the trait of dwarf growth which already characterise this cultivar and should be taken into account for cultivar selection for the BLSSs [50]. Any variations in growth parameters in Space-oriented ecologically closed systems can determine valuable changes in terms of optimisation of volume and resource recycling. These changes can be straightaway converted into economic saving or waste [9]. For example, the increase in the number and size of leaves induced in “Microtom” especially by low doses of irradiation can be beneficial to atmosphere regeneration of pressurised modules, provided that formed leaves do not show structural aberrations which reduce their function.

General anatomical structure of both juvenile and compound “Microtom” leaves was only slightly perturbed in plants from seeds subjected to the highest levels of radiation. Clear-cut dose-depended tendencies of variations were not found in lamina thickness and cell size and shape of the various tissues. The formation of smaller palisade cells was found only in juvenile leaves of irradiated seeds, while an increase in cell size was found only in spongy parenchyma and lower epidermis of compound leaves from seeds irradiated at 100 Gy. The diverse growth behaviour found in the various tissues of structures of different age is in agreement with the different light-mediated control of cell development in the various leaf tissues [51]. Altered mesophyll, characterized by partly collapsed cells with irregular cell shape, was found only sporadically after the exposure of seeds to 50 and 100 Gy in the Microtom cultivar. Such modifications were less marked than those found in leaves of bean where the target organs for irradiation were the leaves themselves [30]; leaves directly exposed to X-rays in bean showed the formation of more loose parenchyma with larger cells, likely due to a radiation-induced damage to cell walls that reduced mechanical constraints to cell enlargement [30, 52]. Further investigations on cell wall composition would be useful to elucidate whether a possible alteration of cell walls was responsible for cell enlargement in the spongy parenchyma and epidermis of leaves from seeds of “Microtom” irradiated with the highest dose of X-rays. However, in the cultivar Microtom, the slight alterations of mesophyll structure did not impair photosynthetic efficiency, as demonstrated by the lack of significant dose-dependent variation trends in the quantum yield of PSII electron transport (ΦPSII), nonphotochemical quenching (NPQ), and maximal PSII photochemical efficiency (Fv/Fm). The increased values of Fv/Fm, close to 0.8 which is considered an optimal value for healthy plants [53], suggested that the photosynthetic apparatus of the leaves from irradiated seeds has higher levels of photochemical efficiency, irrespective of dose. The unchanged or increased content of both chlorophyll and carotenoid pigments confirmed that the photosynthetic apparatus remained stable also in terms of light harvesting capacity and indicated no deleterious effect of radiation on plant photosynthetic machinery. Recently, Marcu et al. [17] showed that irradiation of lettuce seeds with gamma rays up to 30 Gy enhanced the content of chlorophyll a, chlorophyll b, and carotenoids, while higher doses (up to 70 Gy) resulted in their decrease. However, the effect of ionising radiation on photosynthetic pigment content is quite controversial. Increased pigment content is ascribed to the phenomenon of* hormesis*, while a lower content is generally ascribed to the inhibition of chlorophyll and carotenoid synthesis or to oxidation due to the high levels of radiation-induced water radiolysis [2, 14, 54–56]. Some plants possess natural screens against radiation when their tissues contain phenolic compounds with antioxidant functions and can counteract photoinhibitory processes [29, 57]. An increase of phenolic compounds linked to the chloroplast membranes has been recently found in* Phaseolus vulgaris* irradiated leaves exposed to high levels of X-rays [28, 30]. Changes in phenolic content after the exposure to Space can be attributed either to direct effects on the synthesis or mobilization of phenolic compounds or to indirect effects due to changes in ultrastructural organization, such as the increase in the number of chloroplasts per cell [30, 58]. The lowering of phenolic content at high levels of X-rays in “Microtom” leaves is not in agreement with general trends, indicating enhanced phenolics production in plants grown in Space [58] or in various plant tissues directly irradiated with gamma rays [59]. However, the low content of phenolics can be an indirect effect of increasing volume occupied by intercellular spaces due to the loosening of parenchyma cells.

In conclusion, our results show that irradiation of the dry seeds of the Microtom cultivar did not hamper germination and the development of functional leaves. The formation of more compact plants characterised by a higher number of larger leaves can be valuable in terms of increasing resource regeneration in the BLSSs. High levels of radiation induced only slight structural perturbations which were similar in juvenile and compound leaves. Such perturbations did not affect photosynthetic efficiency, since the maximal PSII photochemical efficiency was even increased after irradiation at all doses. However, the tendency to accumulate lower amounts of phenolic compounds along chloroplast membranes after irradiation of seeds with high doses of radiation should be further investigated because it could determine the reduction of natural photoprotection [57]. Finally, the overall results indicate that the Microtom cultivar, irradiated at the seed stage, has high radioresistance during the growth, which is a desirable feature for plant cultivation in Space. Radioresistance, together with dwarf growth and short life cycle, makes this cultivar a valuable candidate for the cultivation in BLSSs.

## Figures and Tables

**Table 1:** Cell shape in different tissues of juvenile leaves of *S. lycopersicum* L. “Microtom”. Mean values ± standard errors are shown (*n* = 75). Different letters correspond to significantly different values according to multiple comparison tests (*P* < 0.05).

Leaf JL		Aspect ratio		Sphericity		Convexity	
Upper epidermis	C	1.97 ± 0.16	a	0.308 ± 0.050	a	0.926 ± 0.021	ab
	0.3 Gy	2.00 ± 0.13	a	0.290 ± 0.038	a	0.941 ± 0.013	a
	10 Gy	2.10 ± 0.12	a	0.239 ± 0.037	a	0.898 ± 0.016	ab
	20 Gy	2.17 ± 0.14	a	0.258 ± 0.045	a	0.909 ± 0.019	abc
	50 Gy	1.70 ± 0.08	a	0.376 ± 0.037	a	0.927 ± 0.016	ab
	100 Gy	1.97 ± 0.14	a	0.286 ± 0.042	a	0.876 ± 0.028	c

Palisade parenchyma	C	2.90 ± 0.10	a	0.109 ± 0.015	a	0.879 ± 0.010	a
	0.3 Gy	3.04 ± 0.12	a	0.099 ± 0.016	a	0.805 ± 0.016	b
	10 Gy	3.09 ± 0.13	a	0.093 ± 0.019	a	0.792 ± 0.015	b
	20 Gy	2.83 ± 0.11	a	0.101 ± 0.017	a	0.784 ± 0.017	b
	50 Gy	3.14 ± 0.14	a	0.082 ± 0.016	a	0.772 ± 0.016	b
	100 Gy	2.75 ± 0.11	a	0.119 ± 0.020	a	0.805 ± 0.017	b

Spongy parenchyma	C	1.90 ± 0.08	a	0.307 ± 0.033	a	0.893 ± 0.012	a
	0.3 Gy	1.80 ± 0.07	a	0.341 ± 0.029	a	0.876 ± 0.013	a
	10 Gy	2.03 ± 0.10	a	0.290 ± 0.031	a	0.901 ± 0.012	a
	20 Gy	1.94 ± 0.12	a	0.344 ± 0.040	a	0.881 ± 0.015	a
	50 Gy	1.74 ± 0.09	a	0.393 ± 0.035	a	0.883 ± 0.014	a
	100 Gy	1.98 ± 0.12	a	0.322 ± 0.037	a	0.878 ± 0.015	a

Lower epidermis	C	1.78 ± 0.09	bc	0.343 ± 0.045	ab	0.919 ± 0.019	a
	0.3 Gy	2.25 ± 0.17	ab	0.263 ± 0.044	b	0.933 ± 0.024	a
	10 Gy	2.19 ± 0.11	abc	0.218 ± 0.034	b	0.888 ± 0.020	ab
	20 Gy	1.80 ± 0.13	bc	0.378 ± 0.047	ab	0.926 ± 0.017	a
	50 Gy	1.67 ± 0.01	c	0.461 ± 0.051	a	0.924 ± 0.021	a
	100 Gy	2.38 ± 0.21	a	0.257 ± 0.057	b	0.857 ± 0.030	b

**Table 2:** Cell shape in different tissues of compound leaves of *S. lycopersicum* L. “Microtom”. Mean values ± standard errors are shown (*n* = 75). Different letters correspond to significantly different values according to multiple comparison tests (*P* < 0.05).

Leaf CL		Aspect ratio		Sphericity		Convexity	
Upper epidermis	C	1.66 ± 0.07	c	0.422 ± 0.042	a	0.946 ± 0.010	a
	0.3 Gy	2.16 ± 0.17	ab	0.244 ± 0.033	c	0.925 ± 0.016	ab
	10 Gy	1.71 ± 0.11	bc	0.401 ± 0.042	a	0.914 ± 0.013	b
	20 Gy	1.86 ± 0.10	abc	0.328 ± 0.041	ab	0.919 ± 0.020	ab
	50 Gy	2.25 ± 0.18	a	0.247 ± 0.041	c	0.897 ± 0.019	b
	100 Gy	1.88 ± 0.09	abc	0.319 ± 0.045	ab	0.902 ± 0.016	b

Palisade parenchyma	C	3.55 ± 0.12	a	0.073 ± 0.014	b	0.890 ± 0.012	a
	0.3 Gy	2.89 ± 0.10	b	0.102 ± 0.013	ab	0.847 ± 0.018	b
	10 Gy	3.00 ± 0.14	b	0.104 ± 0.021	ab	0.826 ± 0.017	b
	20 Gy	3.02 ± 0.11	b	0.094 ± 0.013	ab	0.810 ± 0.014	b
	50 Gy	2.83 ± 0.12	b	0.107 ± 0.021	ab	0.750 ± 0.021	c
	100 Gy	2.75 ± 0.12	b	0.122 ± 0.023	a	0.809 ± 0.015	b

Spongy parenchyma	C	1.93 ± 0.10	a	0.323 ± 0.035	a	0.904 ± 0.011	a
	0.3 Gy	1.83 ± 0.10	a	0.375 ± 0.042	a	0.879 ± 0.013	a
	10 Gy	1.76 ± 0.07	a	0.357 ± 0.034	a	0.869 ± 0.011	a
	20 Gy	1.80 ± 0.09	a	0.366 ± 0.038	a	0.885 ± 0.016	a
	50 Gy	1.71 ± 0.06	a	0.372 ± 0.034	a	0.879 ± 0.015	a
	100 Gy	1.88 ± 0.10	a	0.339 ± 0.037	a	0.883 ± 0.015	a

Lower epidermis	C	1.48 ± 0.08	b	0.545 ± 0.045	a	0.957 ± 0.015	a
	0.3 Gy	2.10 ± 0.17	a	0.295 ± 0.046	b	0.929 ± 0.019	ab
	10 Gy	1.90 ± 0.12	ab	0.334 ± 0.043	b	0.893 ± 0.019	b
	20 Gy	1.78 ± 0.11	ab	0.388 ± 0.047	b	0.929 ± 0.017	ab
	50 Gy	1.72 ± 0.09	ab	0.367 ± 0.040	b	0.914 ± 0.016	b
	100 Gy	1.91 ± 0.14	ab	0.362 ± 0.054	b	0.888 ± 0.025	b
